# The complete chloroplast genome and phylogenetic analysis of *Ophiorrhiza guizhouensis* (Rubiaceae), a folk medicinal plant from the Wuling Mountain region

**DOI:** 10.1080/23802359.2025.2602962

**Published:** 2025-12-18

**Authors:** Gongping Kang, Ning Zhang, Chuandong Yang

**Affiliations:** ^a^ College of Agriculture and Biotechnology, Hunan University of Humanities, Science and Technology, Loudi, China; ^b^ Guizhou Provincial Key Laboratory for Biodiversity Conservation and Utilization in the Fanjing Mountain Region, Tongren, China

**Keywords:** Chloroplast genome, *Ophiorrhiza guizhouensis*, phylogenetic analyses, folk medicinal plant, Wuling Mountain region

## Abstract

*Ophiorrhiza guizhouensis* has not had its complete chloroplast genome reported, which limits our understanding of its genetics and evolution. In this study, we assembled and annotated its chloroplast genome, revealing a circular structure with 80 protein-coding genes (GenBank accession number: PX023271). The total length of the genome was found to be 154,134 bp, and its GC content was 37.76%. Phylogenetic analysis confirmed that *Ophiorrhiza guizhouensis* belonged to the genus *Ophiorrhiza* (subfamily Rubioideae, Rubiaceae) and exhibited the closest phylogenetic affinity to *Ophiorrhiza densa*. These findings provide valuable genomic resources for in-depth studies on *Ophiorrhiza* and the Rubiaceae family, including genetic diversity analysis and phylogenetic research.

## Introduction

**Figure 1.**
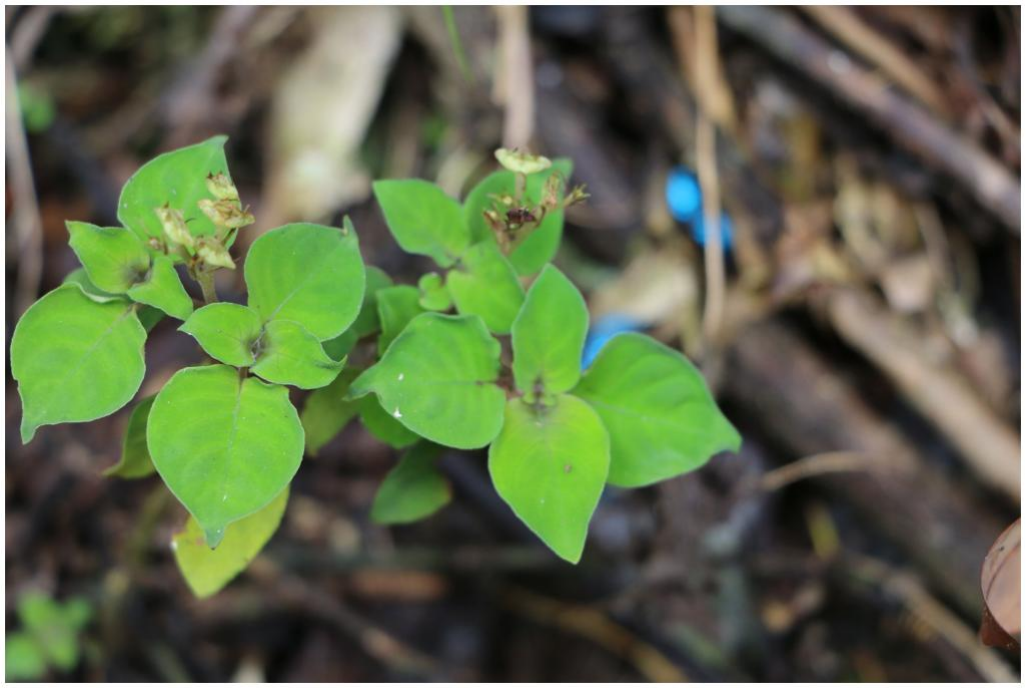
Morphological photograph of *O. guizhouensis* (photographed by Gongping Kang and Chuandong Yang). The morphological characteristics of *O. guizhouensis* include terete stems that are densely hirtellous, usually persistent and ciliate stipules with well-developed colleters on the inner side of the base, as well as shorter corolla tubes, stamens, and styles.

*Ophiorrhiza guizhouensis* Yang, 2018 (Rubiaceae) (*O. guizhouensis*) was discovered in the surrounding areas of Fanjing Mountain within the Wuling Mountain region of China (Yang, He, et al. 2018). The morphological characteristics of *O. guizhouensis* include terete stems that are densely hirtellous, usually persistent and ciliate stipules with well-developed colleters on the inner side of the base, as well as shorter corolla tubes, stamens, and styles (Figure 1). Plants of the *Ophiorrhiza* contain circulation-promoting compounds and have analgesic, antitussive, and mucolytic effects. They are mainly used to treat hemoptysis, bronchitis, and sprains (Yi et al. 2007). The stems and leaves of *Ophiorrhiza japonica*, a species in the same genus, contain camptothecin, which has demonstrated anticancer activity in clinical trials (Lu et al. 2009; Hu et al. 2015). In Western Hunan, China, *O. guizhouensis* is used either in combination or as a substitute for its relative *O. japonica* in traditional folk medicine (Yang, Mou, et al. 2018).

Chloroplast genome research can overcome the limitations of traditional morphological taxonomy and has become a valuable tool for elucidating phylogenetic relationships and the evolutionary trajectories of different species (Raman et al. 2023; Xu et al. 2024; Hou et al. 2025). In this study, we successfully assembled the chloroplast genome of *O. guizhouensis* and elucidated its phylogenetic position within the *Ophiorrhiza* genus. By reporting the first chloroplast genome of *O. guizhouensis*, we aim to provide a foundational genomic resource to promote future comparative genomics and genetics studies of the genus *Ophiorrhiza* and the family Rubiaceae.

## Materials and methods

### Sample collection and genomic DNA extraction

In May 2021, fresh *O. guizhouensis* leaves were collected from Miaowangpo, Dewang Township, Jiangkou County, Guizhou Province (Geographic location, 27°46′31.87″N, 108°33′0.84″E, altitude 868 m). As *O. guizhouensis* is neither an endangered nor a protected species, no special permit is required for its collection. The specimen was deposited in the Plant Specimen Repository of the Key Laboratory for Biodiversity Conservation and Utilization in the Fanjing Mountain Region, Guizhou Province. The specimen (identified and curated by Chuandong Yang, 376709530@qq.com) was assigned the voucher number GZTRUP2021017. Total DNA of *O. guizhouensis* was extracted from young leaves using an optimized CTAB protocol (Abdel-Latif and Osman 2017). Subsequent purity, concentration, and integrity assessments were performed via NanoDrop spectrophotometry and agarose gel electrophoresis.

### Chloroplast genome assembly and annotation

For DNA library construction, a Nextera XT DNA Library Preparation Kit (Illumina Inc., San Diego, CA) was employed, with the resulting library having an average length of 350 bp. Whole genome sequencing was performed on the Illumina NovaSeq 6000 platform (Illumina, Inc., San Diego, CA). High-quality paired-end reads (9.85 Gb) were generated for *O. guizhouensis*. GetOrganelle (v1.7.5) was employed for *de novo* chloroplast genome assembly with default parameters (Jin et al. 2020). Annotation of the chloroplast genome was conducted using CPGAVAS2 (Shi et al. 2019), where the reference genome was *Ophiorrhiza densa* (GenBank accession no. NC_058252.1), and visualization of the chloroplast genome map was conducted with OGDRAW (Greiner et al. 2019). The chloroplast genome’s tRNA genes were identified using tRNAscan-SE (Chen et al. 2015). CPGView (Liu et al. 2023) and Apollo (Lewis et al. 2002) were employed to manually validate and correct erroneous annotations in chloroplast genomes.

### Phylogenetic analysis

Chloroplast genomes used for phylogenetic tree construction were downloaded from the NCBI database. Multiple sequence alignment (MSA) analysis was performed using MAFFT (v7.525) (Katoh and Standley 2013). Subsequently, a maximum-likelihood (ML)-based phylogenetic tree was constructed using IQ-TREE (v2.2.5) (Nguyen et al. 2015) with parameters ‘-alrt 1000 -B 1000’. Finally, the results of the phylogenetic analysis were visualized using iTOL (Letunic and Bork 2019).

## Results

**Figure 2.**
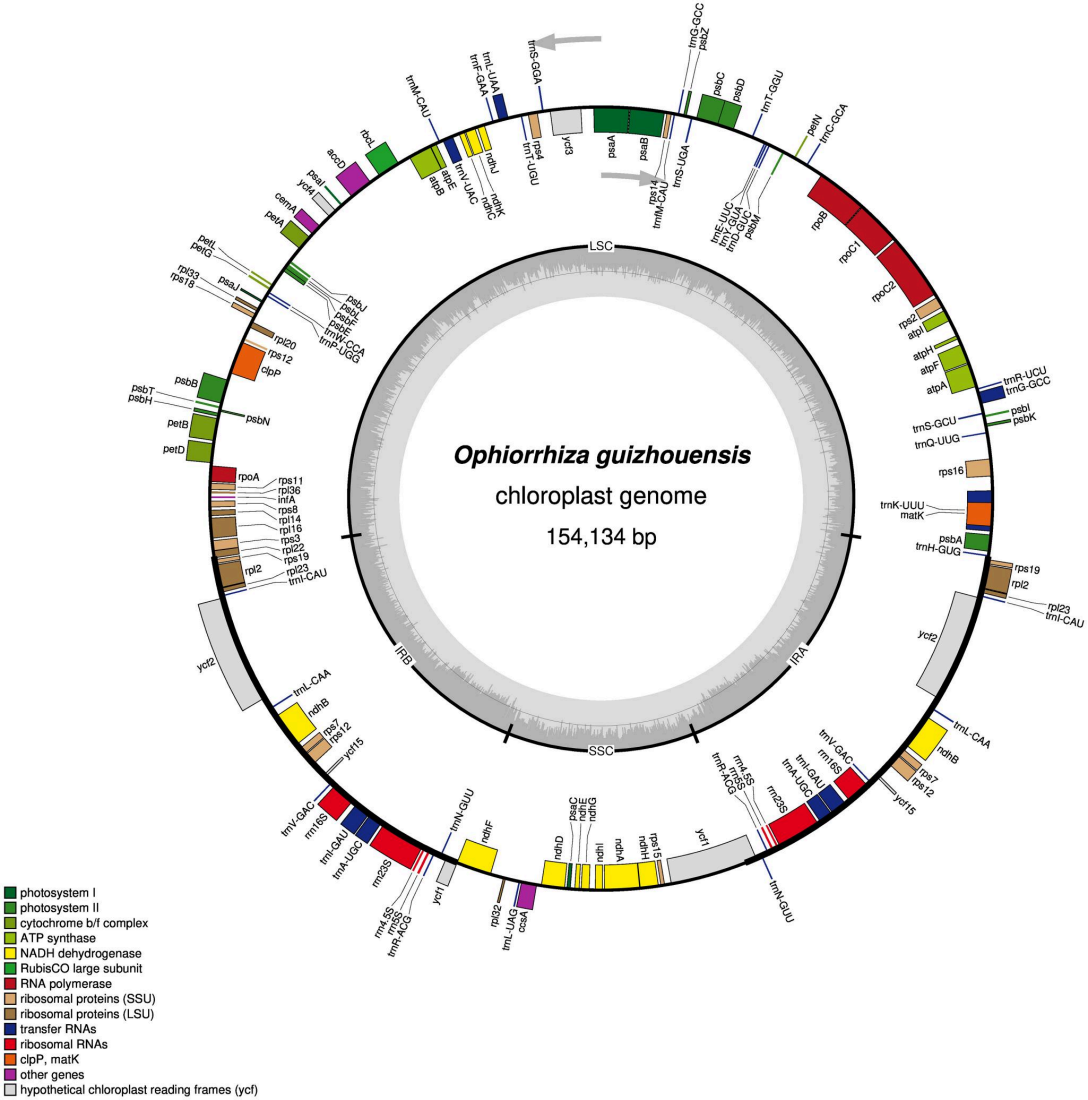
The chloroplast genome map of *O. guizhouensis*. The circular rings in the figure represent the complete chloroplast genome of *O. guizhouensis*. Each colored block corresponds to a specific gene, with different colors indicating distinct functional categories. In the circular map, genes plotted on the outer ring are located on the sense strand, while those on the inner ring are situated on the complementary strand (commonly referred to as the antisense strand). Arrows indicate the transcriptional direction of the genes. The gray area in the inner ring represents the GC content (drawn by OGDRAW (v1.3.1)).

The complete chloroplast genome of *O. guizhouensis* (GenBank accession no. PX023271) spans 154,134 bp with an overall GC content of 37.76% (Table S1). In addition to illustrating the average sequencing depth (1303.84×) of the chloroplast genome of *O. guizhouensis* (Figure S1), two additional figures are provided: Figure S2 shows the map of the cis-splicing genes, and Figure S3 presents the map of the trans-splicing genes. The chloroplast genome comprises three main regions: a large single-copy (LSC) region spanning 84,346 bp with a GC content of 35.63%; a small single-copy (SSC) region spanning 18,482 bp with a GC content of 32.03%; a pair of inverted repeat (IR) regions, each spanning 25,653 bp, with a combined GC content of 43.21% (Figure 2, Table S2).

The chloroplast genome of *O. guizhouensis* encodes 80 unique protein-coding genes (including nine multicopy genes), 29 tRNA genes (eight multicopy), and four rRNA genes (all multicopy) (Table S3). These protein-coding genes comprised 16 gene families, including 11 NADH dehydrogenase subunit genes, five photosystem I subunit genes, 16 photo system II subunit genes, six cytochrome b/f complex subunit genes, six ATP synthase subunit genes, one ribulose-1, 5-bisphosphate carboxylase/oxygenase large subunit gene, four DNA-dependent RNA polymerase genes, nine ribosomal large subunit genes, 12 ribosomal small subunit genes, one mature enzyme gene, one c-type cytochrome synthase gene, one membrane protein gene, one protease gene, one acetyl-CoA-carboxylase subunit gene, one translation initiation factor gene, and four conserved open reading frame genes (Table S3).

**Figure 3.**
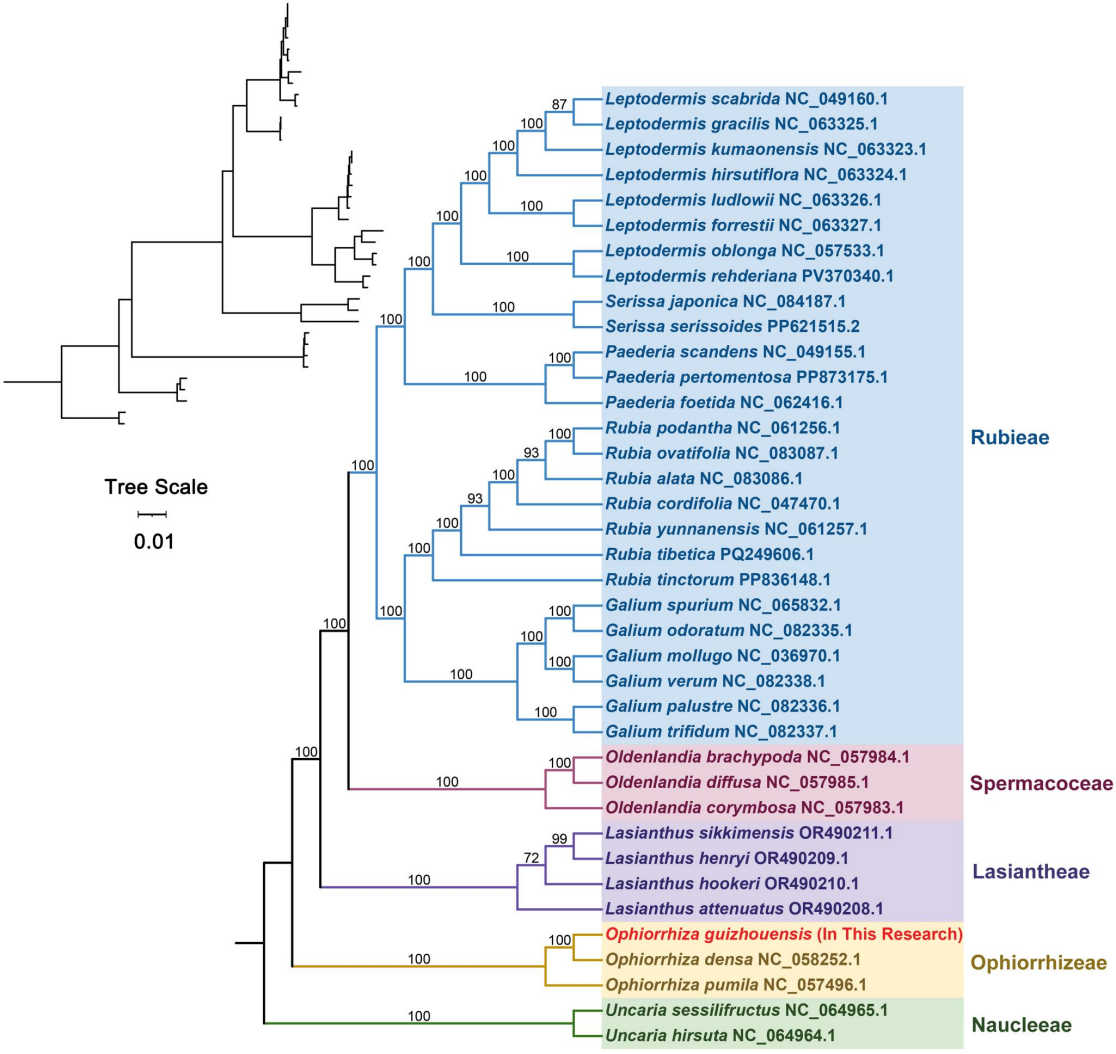
The phylogenetic tree is based on chloroplast genome sequences of *O. guizhouensis* species from the Rubiaceae family. The bar indicates the tree scale, where ‘0.01’ means the length of the black line segment corresponds to 0.01 evolutionary units. Two Rubiaceae species (*Uncaria sessilifructus* and *Uncaria hirsuta*) were used as the outgroups. The species obtained in this study is indicated in red text. The chloroplast sequences used in the analysis are as follows: *Ophiorrhiza densa* NC_058252.1, *Ophiorrhiza pumila* NC_057496.1, *Lasianthus attenuatus* OR490208.1 (Zhang et al. 2024), *Lasianthus henryi* OR490209.1, *Lasianthus hookeri* OR490210.1, *Lasianthus sikkimensis* OR490211.1, *Oldenlandia brachypoda* NC_057984.1, *Oldenlandia corymbosa* NC_057983.1, *Oldenlandia diffusa* NC_057985.1, *Galium mollugo* NC_036970.1 (Dann et al. 2017), *Galium odoratum* NC_082335.1, *Galium palustre* NC_082336.1, *Galium spurium* NC_065832.1 (Yin et al. 2023), *Galium trifidum* NC_082337.1, *Galium verum* NC_082338.1, *Leptodermis forrestii* NC_063327.1 (Zhang et al. 2021), *Leptodermis gracilis* NC_063325.1 (Zhang et al. 2021), *Leptodermis hirsutiflora* NC_063324.1 (Zhang et al. 2021), *Leptodermis kumaonensis* NC_063323.1 (Zhang et al. 2021), *Leptodermis ludlowii* NC063326.1 (Zhang et al. 2021), *Leptodermis oblonga* NC_057533.1 (Guo et al. 2021), *Leptodermis rehderiana* PV370340.1, *Leptodermis scabrida* NC_049160.1 (Zhang et al. 2019) *Paederia foetida* NC_062416.1 (Wang et al. 2022), *Paederia pertomentosa* PP873175.1, *Paederia scandens* NC_049155.1 (Li et al. 2019), *Rubia alata* NC_083086.1 (Shi et al. 2024), *Rubia cordifolia* NC_047470.1, *Rubia ovatifolia* NC_083087.1 (Shi et al. 2024), *Rubia podantha* NC_061256.1, *Rubia tibetica* PQ249606.1 (Li et al. 2025), *Rubia tinctorum* PP836148.1, *Rubia yunnanensis* NC_061257.1 (Zhao et al. 2022), *Serissa japonica* NC_084187.1, *Serissa serissoides* PP621515.2, *Uncaria hirsute* NC_064964.1, and *Uncaria sessilifructus* NC_064965.1.

Based on chloroplast genomes, a phylogenetic tree was constructed for 38 species within the family Rubiaceae (Gentianales). The outgroup used for this analysis comprised two chloroplast genomes from the genus *Nauclea* (subfamily Cinchonoideae, Gentianales). The topological structure of the phylogenetic tree derived from chloroplast genomic data is congruent with the latest classification system proposed by the Angiosperm Phylogeny Group (APG IV). *Ophiorrhiza guizhouensis* belongs to the genus *Ophiorrhiza* (subfamily Rubioideae, Rubiaceae) and exhibits the closest phylogenetic affinity to *Ophiorrhiza densa* (GenBank accession no. NC_058252.1) (Figure 3).

## Discussion and conclusions

This study is the first to annotate and describe chloroplast genome structure of *O. guizhouensis*. The total length of the genome is 154,134 bp, with a GC content of 37.76%. The genome has a typical four-region structure (LSC region, an SSC region, and a pair of IRs) like that of other angiosperms (Gu et al. 2021; Ahmad et al. 2022; Zhang et al. 2024). Phylogenetic analysis indicates that *O. guizhouensis* belongs to the genus *Ophiorrhiza* (subfamily Rubioideae, Rubiaceae) and exhibits the closest phylogenetic affinity to *O. densa*. This is because chloroplast genome sequences are highly conserved. *O. guizhouensis* and *O. densa* belong to the same genus. The high similarity between their chloroplast genomes also indicates the close genetic relationship between the two species. However, there are differences between their chloroplast genomes: the chloroplast genome of *O. guizhouensis* has a length of 154,134 bp, while that of *O. densa* is 154,079 bp, with a length difference of 55 bp between the two genomes. Analysis via BLAST showed that the identity of the two chloroplast genomes is 99.88%. Meanwhile, the two species exhibit significant differences in morphological characteristics. *O. densa* has glabrous stems, caducous stipules, dense leaf veins, and its flowering period is in November (Lo 1990), in contrast, *O. guizhouensis* has stems densely covered with minute hairs, persistent stipules, sparse leaf veins, and its flowering period is in February.

As an important folk medicinal plant in the Wuling Mountain region of China, *O. guizhouensis* warrants further research to promote the development of new drugs and therapeutic strategies for treating various diseases.

## Supplementary Material

Table S1 S2 S3.docx

Figure S1 S2 S3.docx

## Data Availability

The genome sequence data that support the findings of this study are openly available in GenBank of NCBI at https://www.ncbi.nlm.nih.gov/under accession no. PX023271. The associated BioProject, BioSample, and SRA numbers are PRJNA1297444, SAMN50225432, and SRR34776625, respectively.

## References

[CIT0001] Abdel-Latif A, Osman G. 2017. Comparison of three genomic DNA extraction methods to obtain high DNA quality from maize. Plant Methods. 13(1):1. 10.1186/s13007-016-0152-4 28053646 PMC5209869

[CIT0002] Ahmad W et al. 2022. Complete chloroplast genome sequencing and comparative analysis of threatened dragon trees *Dracaena serrulata* and *Dracaena cinnabari*. Sci Rep. 12(1):16787. 10.1038/s41598-022-20304-6 36202844 PMC9537188

[CIT0003] Chen Y, Ye W, Zhang Y, Xu Y. 2015. High speed BLASTN: an accelerated Mega BLAST search tool. Nucleic Acids Res. 43(16):7762–7768. 10.1093/nar/gkv784 26250111 PMC4652774

[CIT0004] Dann M, Bellot S, Schepella S, Schaefer H, Aurélien T. 2017. Mutation rates in seeds and seed-banking influence substitution rates across the angiosperm phylogeny. BioRxiv. 10.1101/156398

[CIT0005] Greiner S, Lehwark P, Bock R. 2019. Organellar Genome DRAW (OGDRAW) version 1.3.1: expanded toolkit for the graphical visualization of organellar genomes. Nucleic Acids Res. 47(W1):W59–W64. 10.1093/nar/gkz238 30949694 PMC6602502

[CIT0006] Gu L, Su T, Luo GL, Hu GX. 2021. The complete chloroplast genome sequence of *Heteropolygonatum ginfushanicum* (Asparagaceae) and phylogenetic analysis. Mitochondrial DNA B Resour. 6(7):1799–1802. 10.1080/23802359.2021.1933636 34104777 PMC8168753

[CIT0007] Guo XM, Wang ZF, Zhang Y, Wang RJ. 2021. Chromosomal-level assembly of the *Leptodermis oblonga* (Rubiaceae) genome and its phylogenetic implications. Genomics. 113(5):3072–3082. 10.1016/j.ygeno.2021.07.012 34246693

[CIT0008] Hou T et al. 2025. The cytonuclear interactions during grapevine domestication. J Integr Plant Biol. 67:1–18. 10.1111/jipb.13968 PMC12498075 40728102

[CIT0009] Hu B, Wang SS, Du Q. 2015. Traditional Chinese medicine for prevention and treatment of hepatocarcinoma: from bench to bedside. World J Hepatol. 7(9):1209–1232. 10.4254/wjh.v7.i9.1209 26019736 PMC4438495

[CIT0010] Jin JJ et al. 2020. GetOrganelle: a fast and versatile toolkit for accurate de novo assembly of organelle genomes. Genome Biol. 21(1):241. 10.1186/s13059-020-02154-5 32912315 PMC7488116

[CIT0011] Katoh K, Standley DM. 2013. MAFFT multiple sequence alignment software version 7: improvements in performance and usability. Mol Biol Evol. 30(4):772–780. 10.1093/molbev/mst010 23329690 PMC3603318

[CIT0012] Letunic I, Bork P. 2019. Interactive tree of life (iTOL) v4: recent updates and new developments. Nucleic Acids Res. 47(W1):W256–W259. 10.1093/nar/gkz239 30931475 PMC6602468

[CIT0013] Lewis SE et al. 2002. Apollo: a sequence annotation editor. Genome Biol. 3(12):RESEARCH0082. 10.1186/gb-2002-3-12-research0082 12537571 PMC151184

[CIT0014] Li X, Mo X, Wang D. 2025. Phylogeny and evolutionary dynamics of the *Rubia* genus based on the chloroplast genome of *Rubia tibetica*. Sci Rep. 15(1):14370. 10.1038/s41598-025-96665-5 40274932 PMC12022247

[CIT0015] Li Z et al. 2019. Characterization of the complete chloroplast genome of *Paederia scandens* (Rubiaceae): a Chinese folk medicinal plant. Mitochondrial DNA B Resour. 4(2):4075–4076. 10.1080/23802359.2019.1691068 33366325 PMC7707732

[CIT0016] Liu S et al. 2023. CPGView: a package for visualizing detailed chloroplast genome structures. Mol Ecol Resour. 23(3):694–704. 10.1111/1755-0998.13729 36587992

[CIT0017] Lo HS. 1990. Taxonomic revision of the Chinese species of *Ophiorrhiza* (Rubiaceae). Bull Bot Res. 10(2):1–82.

[CIT0018] Lu Y et al. 2009. Molecular characterization and expression analysis of a new cDNA encoding strictosidine synthase from *Ophiorrhiza japonica*. Mol Biol Rep. 36(7):1845–1852. 10.1007/s11033-008-9389-y 18987991

[CIT0019] Nguyen LT, Schmidt HA, Von Haeseler A, Minh BQ. 2015. IQ-TREE: a fast and effective stochastic algorithm for estimating maximum-likelihood phylogenies. Mol Biol Evol. 32(1):268–274. 10.1093/molbev/msu300 25371430 PMC4271533

[CIT0020] Raman G et al. 2023. Extensive characterization of 28 complete chloroplast genomes of *Hydrangea* species: a perspective view of their organization and phylogenetic and evolutionary relationships. Comput Struct Biotechnol J. 21:5073–5091. 10.1016/j.csbj.2023.10.010 37867966 PMC10589384

[CIT0021] Shi JZ et al. 2024. The chloroplast genome sequence and phylogenetic analysis of *Rubia alata* wall and *Rubia ovatifolia* Z. Ying Zhang. (Rubiaceae). Mol Biol Rep. 51(1):1140. 10.1007/s11033-024-10046-1 39527330

[CIT0022] Shi L et al. 2019. CPGAVAS2, an integrated plastome sequence annotator and analyzer. Nucleic Acids Res. 47(W1):W65–W73. 10.1093/nar/gkz345 31066451 PMC6602467

[CIT0023] Wang W et al. 2022. The complete chloroplast genome of the medicinal plant *Paederia foetida* L. Mitochondrial DNA B Resour. 7(7):1218–1220. 10.1080/23802359.2022.2087563 35837498 PMC9275490

[CIT0024] Xu X et al. 2024. Twelve newly assembled *Jasmine* chloroplast genomes: unveiling genomic diversity, phylogenetic relationships and evolutionary patterns among *Oleaceae* and *Jasminum* species. BMC Plant Biol. 24(1):331. 10.1186/s12870-024-04995-9 38664619 PMC11044428

[CIT0025] Yang CD, He XZ, Gou GQ. 2018. *Ophiorrhiza guizhouensis* (Rubiaceae), a new species from Guizhou province, southwestern China. PhytoKeys. 95(95):121–126. 10.3897/phytokeys.95.22506 PMC5904563 29674931

[CIT0026] Yang HL, Mou C, Shi LJ, Wang P, Zhang DG. 2018. Four newly recorded species of medicinal plants in Hunan Province. J Jishou Univ (Nat Sci Ed). 39(6):53–55.

[CIT0027] Yi LB, Peng QZ, Tian XR. 2007. Comparative study on camptothecin content in two species of *Ophiorrhiza* from Western Hunan. J Changsha Univ. 21(5):35–36.

[CIT0028] Yin H et al. 2023. Characterization and phylogenetic analysis of the chloroplast genome of *Galium spurium*. Mitochondrial DNA B Resour. 8(3):443–446. 10.1080/23802359.2023.2172971 37006957 PMC10062210

[CIT0029] Zhang Y et al. 2021. Complete chloroplast genomes of *Leptodermis scabrida* complex: comparative genomic analyses and phylogenetic relationships. Gene. 791(1):145715. 10.1016/j.gene.2021.145715 33984444

[CIT0030] Zhang Y et al. 2024. Comprehensive comparative analysis and development of molecular markers for *Lasianthus* species based on complete chloroplast genome sequences. BMC Plant Biol. 24(1):867. 10.1186/s12870-024-05383-z 39285331 PMC11406864

[CIT0031] Zhang Y, Chen S, Wang R. 2019. The complete chloroplast genome of *Leptodermis scabrida* (Rubiaceae): an endemic shrub in Himalaya-Hengduan Mountains. Mitochondrial DNA B Resour. 5(1):169–170. 10.1080/23802359.2019.1698371 33366471 PMC7748826

[CIT0032] Zhao SY, Liang H, Tang PG, Muchuku JK. 2022. A complete chloroplast genome of *Rubia yunnanensis* diels (Rubiaceae), a traditional Chinese herb endemic to China. Mitochondrial DNA B Resour. 7(8):1466–1467. 10.1080/23802359.2022.2107454 35965643 PMC9367649

