# Supplementary material for: The complete chloroplast genome and phylogenetic analysis of Ophiorrhiza guizhouensis (Rubiaceae), a folk medicinal plant from the Wuling Mountain region
Source: Mitochondrial DNA B Resour. 2025 Dec 18;11(1):111–5. doi: 10.1080/23802359.2025.2602962 (PMC12720660; doi:10.1080/23802359.2025.2602962)
Supplement: Table S1 S2 S3.docx [file TMDN_A_2602962_SM4013.docx]

Table S1. The basic information of *O.guizhouensis* chloroplast genome

| Type | Chloroplast genome |
| --- | --- |
| Structure | circular |
| Circular molecular number | 1 |
| Total length | 154134 bp |
| GC content | 37.76 % |

Table S2. The structural information of *O.guizhouensis* chloroplast genome

| Region name | Start | End | Length（bp） | GC content（%） |
| --- | --- | --- | --- | --- |
| LSC | 1 | 84346 | 84346 | 35.63 |
| IR | 84347 | 109999 | 25653 | 43.21 |
| SSC | 110000 | 128481 | 18482 | 32.03 |
| IR | 128482 | 154134 | 25653 | 43.21 |

Table S3. Classification of genes in the *O.guizhouensis* chloroplast genome

| Group of genes | Name of genes |
| --- | --- |
| Subunits of NADH-dehydrogenase | *ndh*A，*ndh*B(×2)，*ndh*C，*ndh*D，*ndh*E，*ndh*F，*ndh*G，*ndh*H，*ndh*I，*ndh*J，*ndh*K |
| Subunits of photosystem Ⅰ | *psa*A，*psa*B，*psa*C，*psa*I，*psa*J |
| Subunits of photosystem Ⅱ | *psb*A，*psb*B，*psb*C，*psb*D，*psb*E，*psb*F，*psb*H，*psb*I，*psb*J，*psb*K，*psb*L, *psb*M，*psb*N，*psb*T，*psb*Z，*ycf*3 |
| Subunits of cytochrome b/f complex | *pet*A，*pet*B，*pet*D，*pet*G，*pet*L，*pet*N |
| Subunits of ATP synthase | *atp*A，*atp*B，*atp*E，*atp*F，*atp*H，*atp*I |
| Large subunit of rubisco | *rbc*L |
| Small subunit of ribosome | *rps*2，*rps*3，*rps*4，*rps*7(×2)，*rps*8，*rps*11，*rps*12(×2)，*rps*14，*rps*15，*rps*16，*rps*18, *rps*19(×2) |
| Large subunit of ribosome | *rpl*2(×2)，*rpl*14，*rpl*16，*rpl*20，*rpl*22，*rpl*23(×2)，*rpl*32，*rpl*33，*rpl*36 |
| DNA dependent RNA polymerase | *rpo*A，*rpo*B，*rpo*C1，*rpo*C2 |
| rRNA genes | *rrn*4.5S(×2)，*rrn*5S(×2)，*rrn*16S(×2)，*rrn*23S(×2) |
| tRNA genes | *trn*A-UGC(×2)，*trn*C-GCA，*trn*D-GUC，*trn*E-UUC，*trn*F-GAA，*trnf*M-CAU，*trn*G-GCC(×2)，*trn*H-GUG，*trn*I-CAU(×2)，*trn*I-GAU(×2)，*trn*K-UUU，*trn*L-CAA(×2)，*trn*L-UAA，*trn*L-UAG，*trn*M-CAU，*trn*N-GUU(×2)，*trn*P-UGG，*trn*Q-UUG，*trn*R-ACG(×2)，*trn*R-UCU，*trn*S-GCU，*trn*S-GGA，*trn*S-UGA，*trn*T-GGU，*trn*T-UGU，*trn*V-GAC(×2)，*trn*V-UAC，*trn*W-CCA，*trn*Y-GUA |
| Maturase | *mat*K |
| c-type cytochrom synthesis gene | *ccs*A |
| Envelope membrane protein | *cem*A |
| Translational initiation factor | *inf*A |
| Protease | *clp*P |
| Subunit of Acetyl-CoA-carboxylase | *acc*D |
| Genes of unknown functions Open Reading | *ycf*1(×2)，*ycf*2(×2)，*ycf*4，*ycf*15(×2) |

Notes: Gene (2): Number of copies of multi-copy genes.
