# Supplementary material for: The complete chloroplast genome and phylogenetic analysis of Ophiorrhiza guizhouensis (Rubiaceae), a folk medicinal plant from the Wuling Mountain region
Source: Mitochondrial DNA B Resour. 2025 Dec 18;11(1):111–5. doi: 10.1080/23802359.2025.2602962 (PMC12720660; doi:10.1080/23802359.2025.2602962)
Supplement: Figure S1 S2 S3.docx [file TMDN_A_2602962_SM4012.docx]

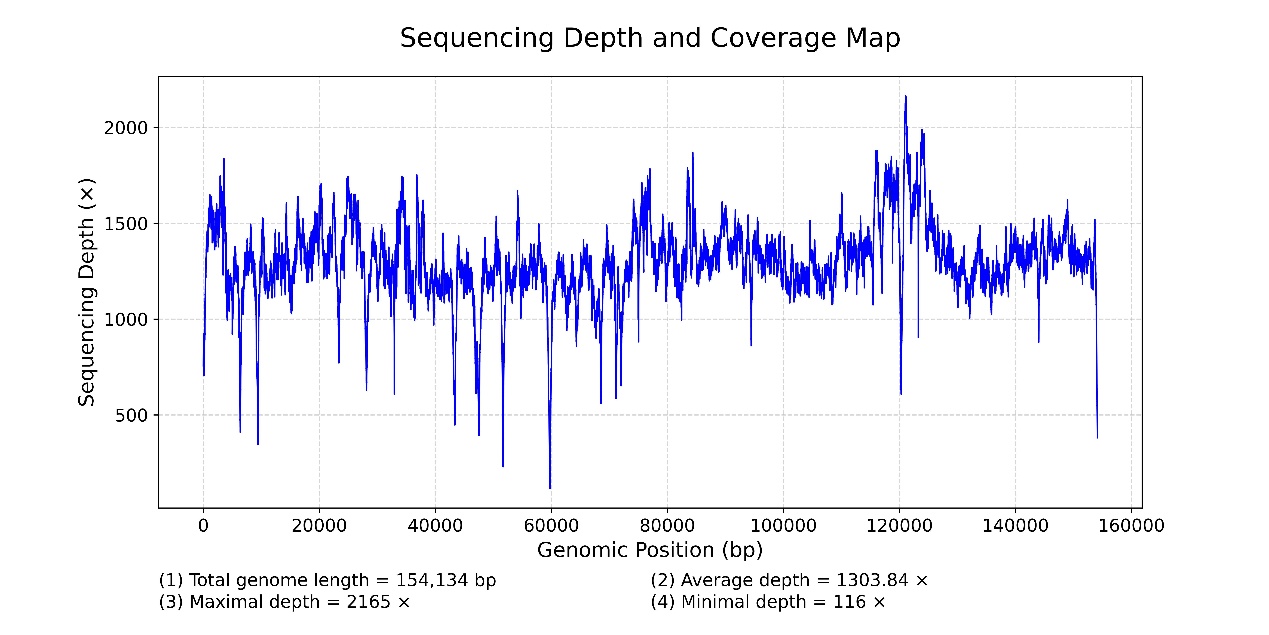


Figure S1. The average sequencing depth of the chloroplast genome of *O. guizhouensis*


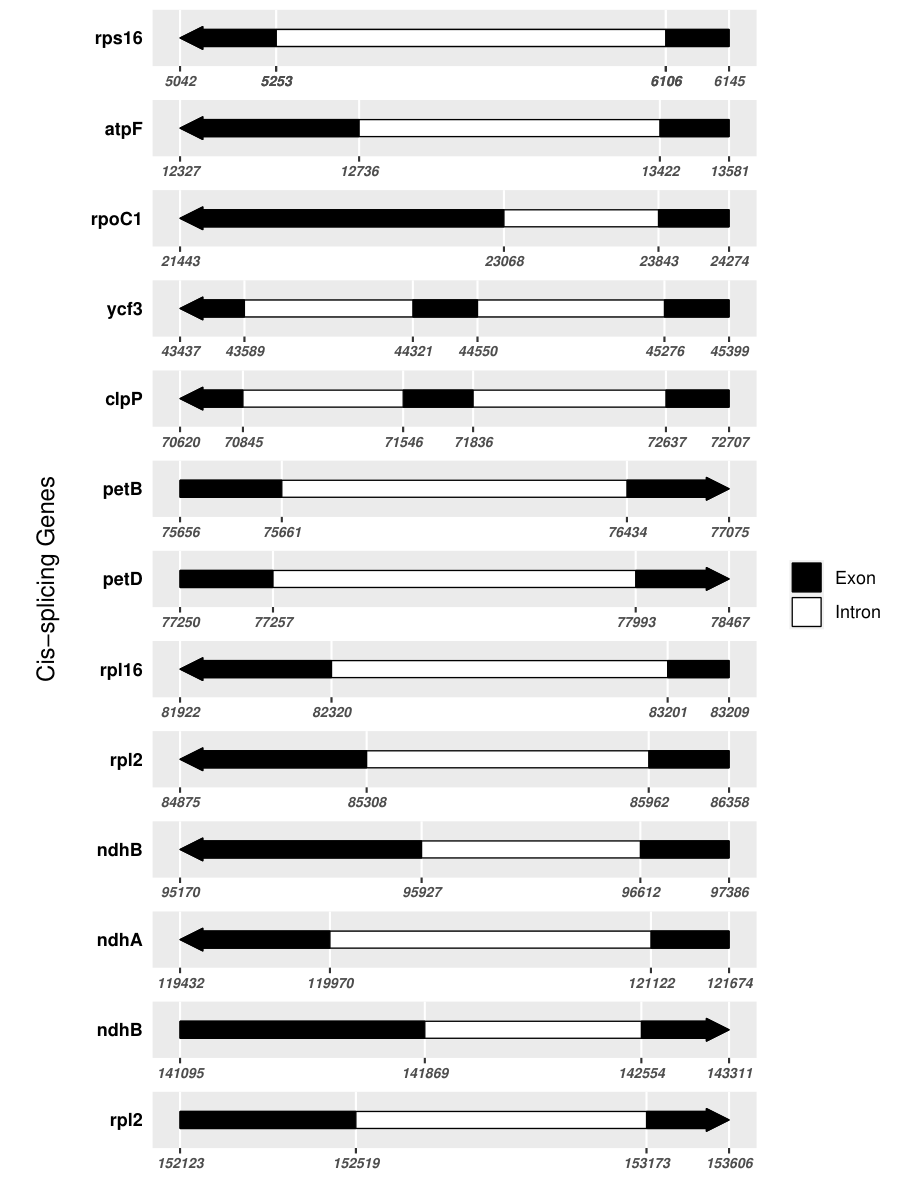


Figure S2. The cis-splicing genes of the chloroplast genome of *O. guizhouensis*


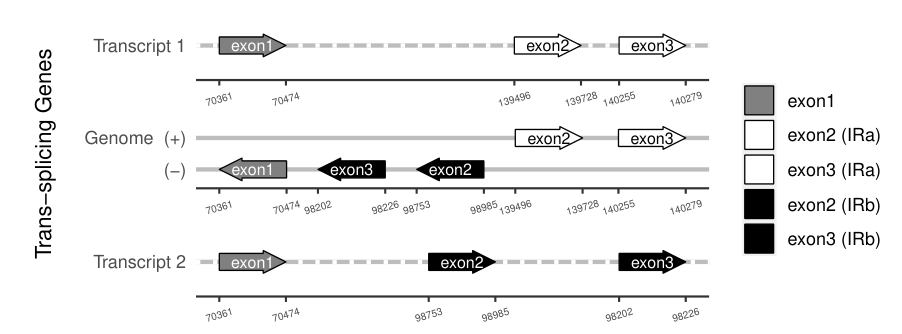


Figure S3. The trans-splicing genes of the chloroplast genome of *O. guizhouensis*
